# An outlier approach: advancing diagnosis of neurological diseases through integrating proteomics into multi-omics guided exome reanalysis

**DOI:** 10.1038/s41525-025-00493-5

**Published:** 2025-05-03

**Authors:** Martin Man-Chun Chui, Anna Ka-Yee Kwong, Hiu Yu Cherie Leung, Chingyiu Pang, Ines F. Scheller, Sheila Suet-Na Wong, Cheuk-Wing Fung, Vicente A. Yépez, Julien Gagneur, Christopher Chun-Yu Mak, Brian Hon-Yin Chung

**Affiliations:** 1 Department of Paediatrics and Adolescent Medicine, School of Clinical Medicine, Li Ka Shing Faculty of Medicine, The University of Hong Kong, Hong Kong SAR, China; 2 School of Computation, Information and Technology, Technical University of Munich, Munich, Germany; 3 Department of Paediatrics and Adolescent Medicine, Hong Kong Children’s Hospital, Hong Kong SAR, China; 4 Institute of Human Genetics, School of Medicine, Technical University of Munich, Munich, Germany

**Keywords:** Bioinformatics, Next-generation sequencing, RNA sequencing, Clinical genetics, Neurological disorders, Neurodevelopmental disorders, Paediatric neurological disorders, Genetics research, Paediatric research

## Abstract

Neurodevelopmental disorders (NDDs) often have unknown genetic causes. Current efforts in identifying disease-related genetic variants using exome or genome sequencing still lead to an excessive number of variants of uncertain significance (VUS). There is an increasing interest in transcriptomics and, more recently, proteomics for variant detection and interpretation. In this study, we integrated quantitative liquid chromatography-mass spectrometry proteomics, RNA sequencing, and exome reanalysis to resolve VUS and detect novel causal variants in 34 patients with undiagnosed NDDs, using the software PROTRIDER and DROP to detect protein outliers and RNA outliers, respectively. We obtained a diagnosis in 11 cases (32%) resulting from the increased amount of information provided by the two additional levels of omics (*n* = 5) and the updated literature evidence (*n* = 6). Our experience suggests the potential of this outlier-detection multi-omics workflow for improving diagnostic yield in NDDs and other rare disorders.

## Introduction

Neurodevelopmental diseases (NDDs) are chronic disorders categorized by similar developmental deficits in cognition, motor, behavior, communication, and social abilities. They present highly variable phenotypes and different ages of onset^1,2^. Pediatric neurological diseases present a significant global burden^3^, and challenges in the clinical diagnosis in children are exacerbated by nonspecific presentations, as phenotypes are often masked by children’s development^1,4^. The identification of precise genetic etiology is, hence, of paramount importance to accurately diagnose and facilitate personalized clinical management and precision treatments^5^. On top of karyotyping and gene-panel microarrays, next-generation sequencing has expanded our ability to interrogate the genomes of patients with rare and difficult-to-diagnose neurological conditions^6,7^. Meta-analyses on the clinical utility of whole-exome sequencing (WES) and whole-genome sequencing (WGS) as first-line diagnostic methods in neurological disease studies estimated a diagnostic yield of 35%–40%^8,9^. Despite this, a large proportion of patients remain undiagnosed; inconclusive genetic diagnoses may hinder patients’ access to effective clinical management and treatment.

Although reanalysis of DNA sequencing data is able to increase the diagnostic yield by approximately 12% through utilizing latest gene-disease association databases and updated clinical phenotypes from recent clinical reviews^10,11^, one of the most prominent challenges remains the interpretation of variants of uncertain significance (VUS)^12^, with studies showing that VUS contributes to 18%–28% of genetically undiagnosed cases^8,9^. These VUS are either not prioritized by current genomic analysis pipelines or lack supporting evidence that suggests their pathogenicity. This challenge is even more pronounced in non-European populations due to the current lack of data on the allelic frequencies within these populations^9^. Interpreting the impact of VUS requires further analysis at levels beyond DNA. RNA sequencing (RNA-seq) has emerged as a companion diagnostic tool that provides functional evidence to interpret VUS by detecting aberrant RNA phenotypes^13,14^. It has been recently applied for genetic diagnosis in different Mendelian diseases, with studies presenting an incremental diagnostic yield of 7.5%–36% in cases unresolved by WES or WGS^15–21^. Multiple studies that employ RNA-seq for the diagnosis of genetic disease utilize what is known as an ‘outlier’ approach, which consists of identifying abnormal RNA expression levels or sequences unique in each patient. Among these approaches, the Detection of RNA Outliers Pipeline (DROP) is a modular computational workflow that integrates the preprocessing of RNA-seq data with statistical algorithms, comprising of OUTRIDER^22^ which detects aberrant expression (AE), FRASER^23^^,24^ which detects aberrant splicing (AS), and a module that detects for monoallelic expression (MAE) in RNA^25^. These are taken as aberrant RNA phenotypes potentially attributable to the presence of pathogenic variants^19,20^. To detect outliers, OUTRIDER and FRASER adopt a denoising autoencoder approach to automatically control for confounders.

While RNA-seq data provides a substantial incremental yield (16% on median) in diagnosing WES/WGS unresolved cases^20^, it only targets variants that might lead to an RNA phenotype. An estimated 26% of VUS could have potential RNA phenotypes based on data retrieved from ClinVar (Supplementary Fig. 1), consisting of 5′-UTR (9%), frameshift (5%), splice donor/acceptors (2%), stop gain (4%), duplication (10%), deletion (6%) and some exonic single-nucleotide variants estimated to induce splice disruption (64%)^12^. The interpretation of the remaining portion of VUS with no RNA phenotypes would be unlikely to benefit from RNA-seq, yet they may still influence protein phenotypes. Variants that can impact protein levels include missense variants, which affect protein stability, nonsense-mediated mRNA decay (NMD)-escaping premature termination codons, and variants affecting post-translational modification (PTM) machineries, which can alter PTM of other proteins and affect their protein stability^26,27^. Proteomics can be used to detect abnormal protein levels resulting from such protein phenotypes. Yet, the role of proteomics in clinical diagnostics has mostly been limited to elucidating the underlying disease pathophysiology^28^, providing cumulative functional evidence to support variants identified by RNA-seq in selected cases^15,29^, or resolving a limited number of cases by guiding targeted genetic testing^30^. Despite having potential diagnostic utility, there has been a lack of studies on the systematic integration of proteomics. A recent study systematically integrated liquid chromatography-mass spectrometry (LC-MS)-based quantitative proteomics with genomics and transcriptomics^31^. They proposed a novel bioinformatics pipeline, PROTRIDER, to be integrated with DROP for the resolution of genetically undiagnosed mitochondrial disorder cases. PROTRIDER uses an outlier approach, similar to OUTRIDER and FRASER, to detect aberrant protein expression^31^^,32^. Demonstrating an incremental diagnostic yield of 22%, this study also showcased the ability of a proteomics approach to provide insights into the impact of missense variants and in-frame indels VUS that cannot be interpreted by RNA-seq^31^.

**Fig. 1:**
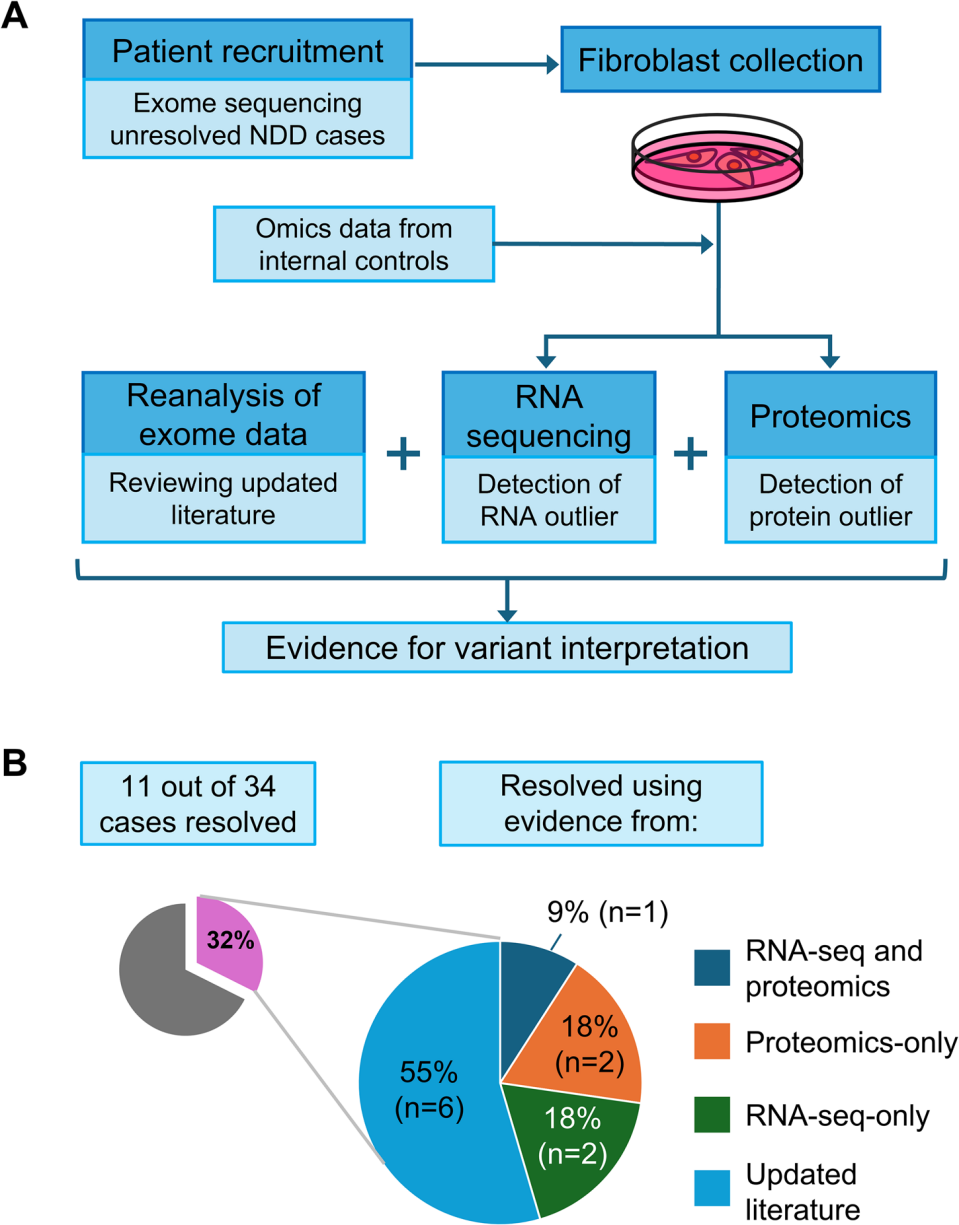
Overview of the study. **A** Diagnostic workflow of our multi-omics pipeline incorporating exome reanalysis, transcriptomics, and proteomics. **B** Diagnostic yield of this study. The pie chart on the left shows the proportion of recruited cases that were resolved in this study. The pie chart on the right shows the number of resolved cases whose resolution was guided by different types of omics analysis. RNA-seq and proteomics: cases where the evidence was found in both RNA-seq and proteomics data. Proteomics-only: Cases where the evidence was found only in proteomics data. RNA-seq-only: Cases where evidence was found only in RNA-seq data. Updated literature: Cases resolved through exome reanalysis using evidence from up-to-date literature, in which 5 cases involved missense variants that may alter protein functions but not affect RNA or protein levels significantly, and hence not assessed by our multi-omics approach for detecting expression outliers.

Extending the application of the multi-omics pipeline pioneered on mitochondrial diseases, we adopted a diagnostic workflow by incorporating it with exome reanalysis in a genetically undiagnosed cohort of 34 patients with NDDs (Fig. 1). Skin fibroblasts were sampled from the recruited participants for RNA and protein extraction owing to its higher coverage of Mendelian disease-associated genes when compared to other clinically accessible tissues^20^. After conducting RNA-seq and LC-MS-based quantitative proteomics, the output data were analyzed using DROP and PROTRIDER, respectively, to detect aberrant events. Our multi-omics workflow increased the overall diagnostic rate of the 34 unresolved NDD cases, and also provided insights into disease mechanisms and potential drug targets, indicating its potential for further facilitating genetic diagnosis and clinical management.

## Results

### Patient demographics

Thirty-four undiagnosed patients with pediatric-onset NDDs (22 males, 12 females) were recruited for this study. The participants were aged between 3 months to 39 years at the time of analysis. The onset of the disorders ranged from neonatal to 7 years of age. Within the time of disease onset to recruitment in this study, the patients had received extensive clinical investigations and genetic testing, including WES (except for SF289, which used targeted genes analysis by NGS/Multiplex ligation-dependent probe amplification), but remained etiologically undiagnosed. In this cohort, 85.3% (29/34) of patients exhibit global developmental delay or intellectual disability, 67.6% (23/34) have developed movement disorders, 41.2% (14 out of 34) experience epilepsy, and 11.8% (4/34) have autism spectrum disorders. The clinical features are summarized in Supplementary Data 1.

### Inclusion of controls

Thirty-five in-house fibroblast RNA-seq samples and 76 publicly available RNA-seq samples from skin fibroblasts of presumed healthy individuals downloaded from the Genotype-Tissue Expression (GTEx) project^33^ were included as controls in the pipeline for RNA outlier detection. For protein-outlier detection, 26 in-house fibroblasts proteomics samples were included as controls.

### Genes covered and aberrant outliers detected by RNA-seq and proteomics analysis

**Fig. 2:**
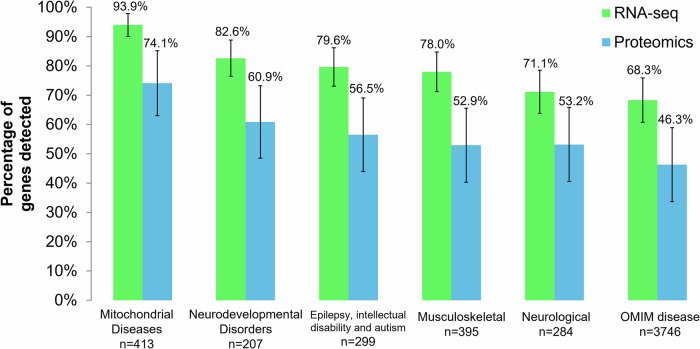
Percentage of genes from different disease gene panels that were detected by RNA-seq and proteomics. The disease gene panels were retrieved from publicly available databases and from the literature. *n* = number of genes in the disease gene panel. Error bars indicate the 95% confidence intervals for each percentage of genes detected.

In total, 17,163 genes and 7278 proteins were detected through RNA-seq and proteomics in the fibroblast samples of the patients and controls. To assess the ability of fibroblast RNA-seq and proteomics to detect relevant disease gene events, we analyzed the coverage of transcripts and proteins in major disease gene panels, including a mitochondrial disease panel^34^, neurodevelopmental disorder panel (https://www.ncbi.nlm.nih.gov/gtr/tests/597367/overview, retrieved on October 2023), intellectual disability and autism panel (ClinGen expert panels), epilepsy panel (ClinGen expert panels), neurological panel^18^, musculoskeletal panel^18^ and OMIM disease panel (https://www.omim.org/). Our transcriptomics pipeline detected 68%–94% of the gene products from these selected panels, while quantitative proteomics detected 46%–74% (Fig. 2). The mitochondrial disease panel (*n* = 413) has the highest coverage from both readouts, followed by neurodevelopmental disorders (*n* = 207). For the ‘neurological disease’ panel specifically, expressed transcripts and proteins covered 71.1% and 53.2%, respectively. This data showed that our transcriptomics and proteomics workflow was both capable of detecting relevant major disease genes from fibroblast samples and can provide additional evidence in aiding the exome reanalysis of unresolved cases.

For RNA-seq analysis, a total of 136, 151, and 3670 outliers (averaging at 4, 4.4, and 111.2 per patient) were detected by AE, AS, and MAE modules, respectively, in the fibroblast sample of our patients (Supplementary Data 1). MAE hits were further prioritized by filtering for rare variants (gnomAD allele frequency <0.01), which halved the number of hits (total 1286; 38.9 per patient). As for proteomics, a total of 58 (1.7 per patient) protein outliers were detected. Filtering the outlier hits for OMIM genes (https://omim.org/, retrieved August 2024), we saw that DROP detected a total of 24, 53, and 140 events (averaging at 0.7, 1.6, and 4.2 per patient) for AE, AS and MAE (for MAE, gnomAD allele frequency <0.01) respectively. Proteomics detected a total of 20 OMIM gene outlier events, averaging 0.59 outliers per patient. This prioritization resulted in a handleable number of variants for manual curation.

### Diagnoses made by multi-omics guided exome reanalysis

Through this study, 11 out of the 34 undiagnosed individuals (32.4%) with neurological disorders received a diagnosis (Table 1). Among these, the identification of genetic defects (in *MSTO1, RNU7-1, GFM1, GARS1*, and *SHMT2*) in 5 individuals (15%) was guided by the detection of significantly aberrant RNA or protein phenotypes by an outlier approach using RNA-seq and proteomics (Table 1). Protein outliers were detected using an adaptation of OUTRIDER for proteomics, PROTRIDER^31^^,32^. We describe here how the diagnoses of these five cases were aided by the outlier approach to end the long diagnostic odysseys of our participants.

**Table 1:** Clinical characteristics and causative variants identified in 11 participants diagnosed by further exome reanalysis

Participant	Gene	Genomic variant	Variant category	Inheritance	Associated disease/ inheritance	Classification / ACMG/AMP or other evidence on pathogenicity	Variant curatable using purely exome reanalysis?	Evidence of variant and pathogenicity that was provided by transcriptomics and proteomics (if any)
SF074	*FBXO28*	Heterozygous NM_015176.4: c.1073_1074del, p.(Leu358GlnfsTer4)	Frameshift (escape NMD)	De novo	DEE100 / AD	Likely pathogenic / PM1, PM4, PM2_supporting, PM6_supporting	Yes	Variant also detected in RNA-seq data. No significant reduction in gene expression was detected as expected for a frameshift variant in the last exon, which escapes NMD.
SF108	*GNB1*	Heterozygous NM_002074.5: c.239T>C, p.(Ile80Thr)	Missense	De novo	Intellectual developmental disorder / AD	Likely pathogenic / PS4_moderate, PM1, PM5, PM2_supporting	Yes	Variant also detected in RNA-seq data.
SF113	*KCNT1*	Heterozygous NM_020822.2: c.1546A>G, p.(Met516Val)	Missense	De novo	DEE14 / AD	Likely pathogenic / PM6_strong, PS4_moderate, PM2_supporting	Yes	Variant also detected in RNA-seq data
SF171	*GABRG2*	Heterozygous NM_00816.3: c.316G>A, p.(Ala106Thr)	Missense	De novo	DEE74 / AD	Likely pathogenic / PS4_moderate, PM6_moderate, PM2_supporting, PP2	Yes	Variant also detected in RNA-seq data
SF180	*ATP1A3*	Heterozygous NM_152296.5: c.2324C>T, p.(Pro775Leu)	Missense	De novo	*ATP1A3*-related disorder / AD	Likely pathogenic / PM5, PS4_moderate, PS3_supporting, PM2_supporting, and PM6_supporting	Yes	None; *ATP1A3* is not expressed in fibroblasts
SF185	*MSTO1*	Compound heterozygous NM_018116.4: c.967-3C>A; c.971C>T, p.(Thr324Ile)	Splice region, missense	Maternal & Paternal	Myopathy, mitochondrial, and ataxia/ AD or AR	Both likely pathogenic / PM3_strong, PM2_supporting, PP4 (for c.971C>T) / PVS1_RNA^40^, PM2_supporting (for c.967-3C>A)	No	RNA-seq revealed unbalanced allele levels. This finding guided the identification of a splice region variant. Reinspection of the RNA expression and splicing results further upgraded it from VUS to likely pathogenic.
SF188	*RNU7-1*	Compound heterozygous NR_023317: n.[28C>T]; [35G>A]	Small nuclear RNA	Maternal & Paternal	Aicardi–Goutières syndrome 9 / AR	Both likely pathogenic / PP4_strong, PM3, PM2_supporting (for both variants)	No	RNA-seq uncovered overexpression of 26 histone genes and guided the identification of *RNU7-1* variants
SF196	*SPAST*	Heterozygous NM_014946.4: c.1385A>G, p.Lys462Arg	Missense	Not available	Spastic paraplegia type 4 / AD	Likely pathogenic / PM1, PM5, PM2_supporting, PP3_moderate	Yes	Variant also detected in RNA-seq data
SF197	*GFM1*	Homozygous (104.5kb deletion chr3:158435847-158540317) covering candidate *GFM1* enhancers	Large genomic deletion	Maternal & Paternal	Combined oxidative phosphorylation deficiency 1 / AR	VUS / Aberrant *GFM1* expression and the identification of candidate *GFM1* enhancers in the deleted region support the damaging effect of the deletion	No	Underexpression outlier identified in both RNA-seq and proteomics, which guided the identification of the deletion.
SF231	*GARS1*	Heterozygous NM_002047.4: c.258_259insGTGGCTGAGCTCAAAGC, p.Pro87ValfsTer9	Frameshift	Maternal	Spinal muscular atrophy, infantile, James type / AD	Likely pathogenic / PVS1, PM2_supporting	Yes	Initially missed by exome analysis. Proteomics guided the identification of the variant, and the insertion was subsequently confirmed in WES and RNA-seq data.
SF269	*SHMT2*	Compound heterozygous NM_005412: c.1042CT, p.R348W & c.1301G>A, p.R434Q	Missense, missense	Maternal & Paternal	Neurodevelopmental disorder with cardiomyopathy, spasticity, and brain abnormalities / AR	Both likely pathogenic / PS3, PM2_supporting, PP4 (for c.1042C>T) / PS3, PM2_supporting, PP4, PP3_moderate (for c.1301G>A),	No	Proteomics guided the identification of the variants and provided functional evidence to upgrade them from VUS to likely pathogenic

*AD* autosomal dominant, *ASD* autism spectrum disorder, *DEE* developmental and epileptic encephalopathy, *WES* whole-exome sequencing, *GDD* global developmental delay, *ID* intellectual disability, *LL* lower limb, *NMD* nonsense-mediated decay, *VUS* variant of uncertain significance.

**Fig. 3:**
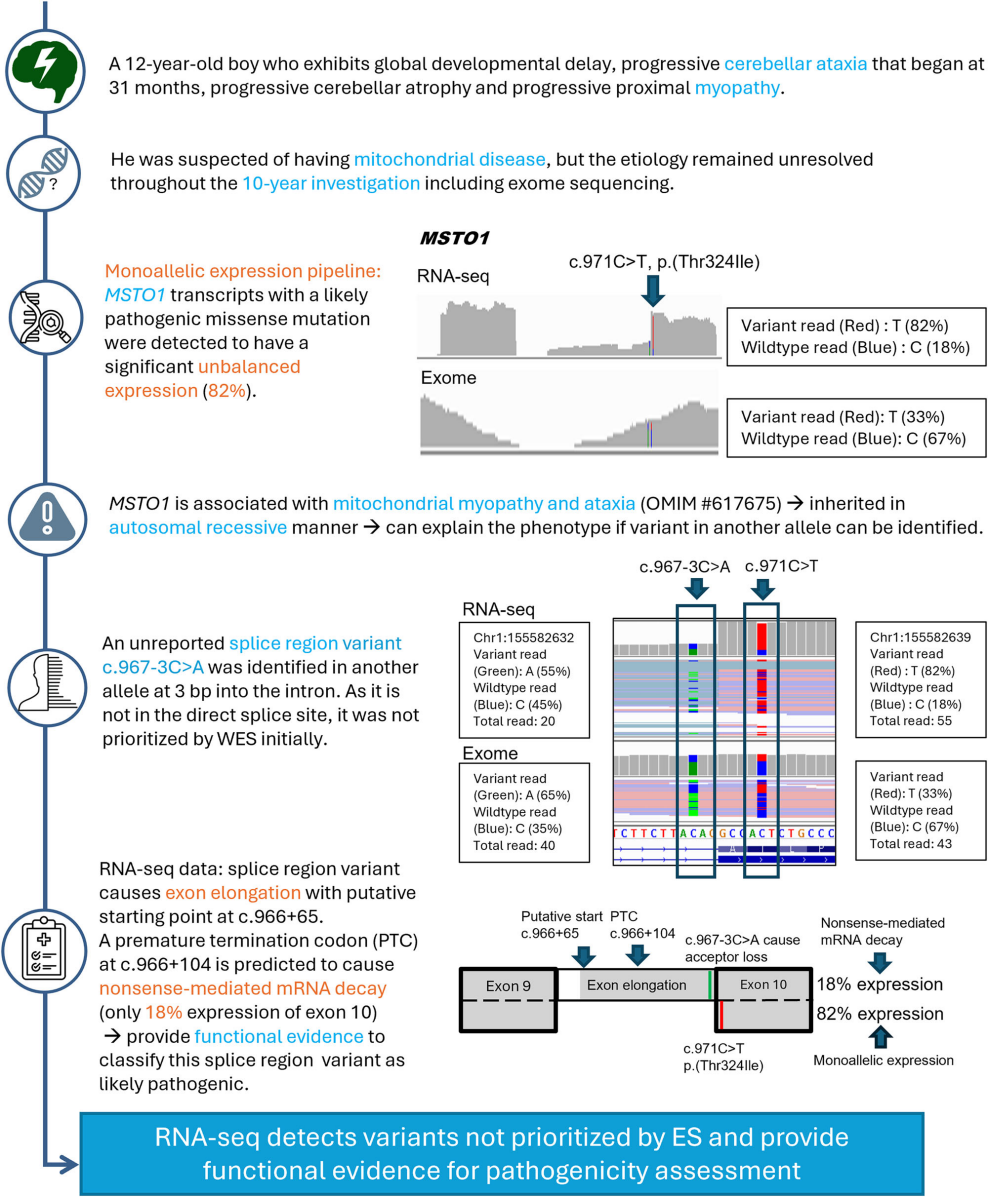
Identification of the splice region *MSTO1* variant in participant SF185. The MAE pipeline detected that one of the alleles with a missense variant c.971C>T in *MSTO1* has significant monoallelic expression (82%), suggesting a variant triggering nonsense-mediated decay in the other allele. A heterozygous splice region variant c.967-3C>A was identified in the other allele, leading to exon elongation with a putative starting point at c.966+65 and a premature termination codon at c.966+104. Reinspection of the expression and splicing results led to the finding that the exon elongation is aberrant (nominal *p*-value of 0.0258 and an effect size Δ*J* of −0.2) and causes a global reduction in expression (OUTRIDER: fold change = 0.71, Z-score = −4.13, nominal *p*-value = 1.13 × 10^−4^). This case highlights the importance of RNA-seq results reevaluation guided by genetic or clinical data, as for both expression and splicing analyses, the FDR was above the significance cutoff of 0.1.

**Fig. 4:**
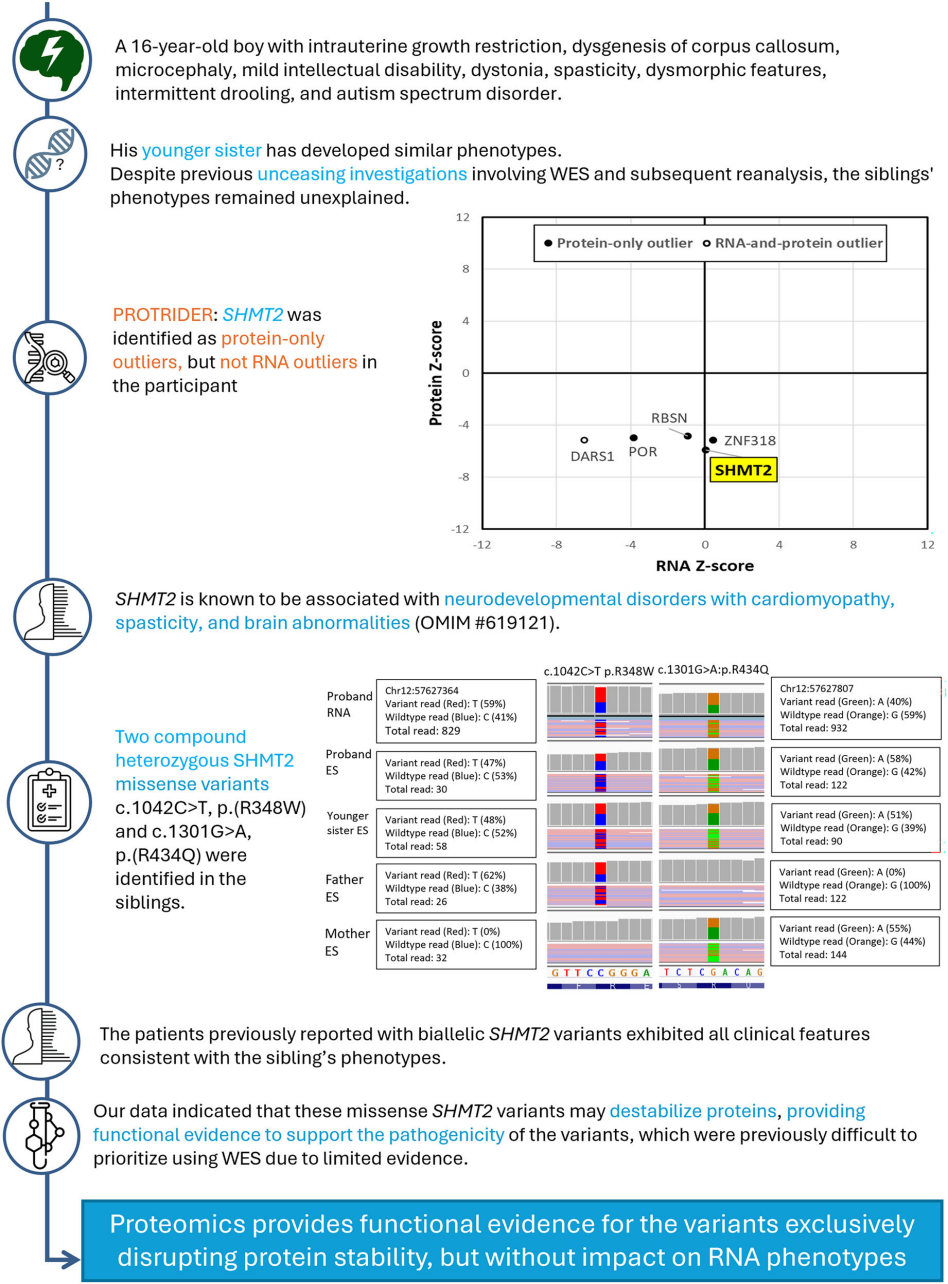
*SHMT2* protein-only outlier in participant SF269. Detection of *SHMT2* protein-only outlier guided the identification of two compound heterozygous *SHMT2* missense variants, which lead to protein destabilization but not transcriptional dysregulation.

**Fig. 5:**
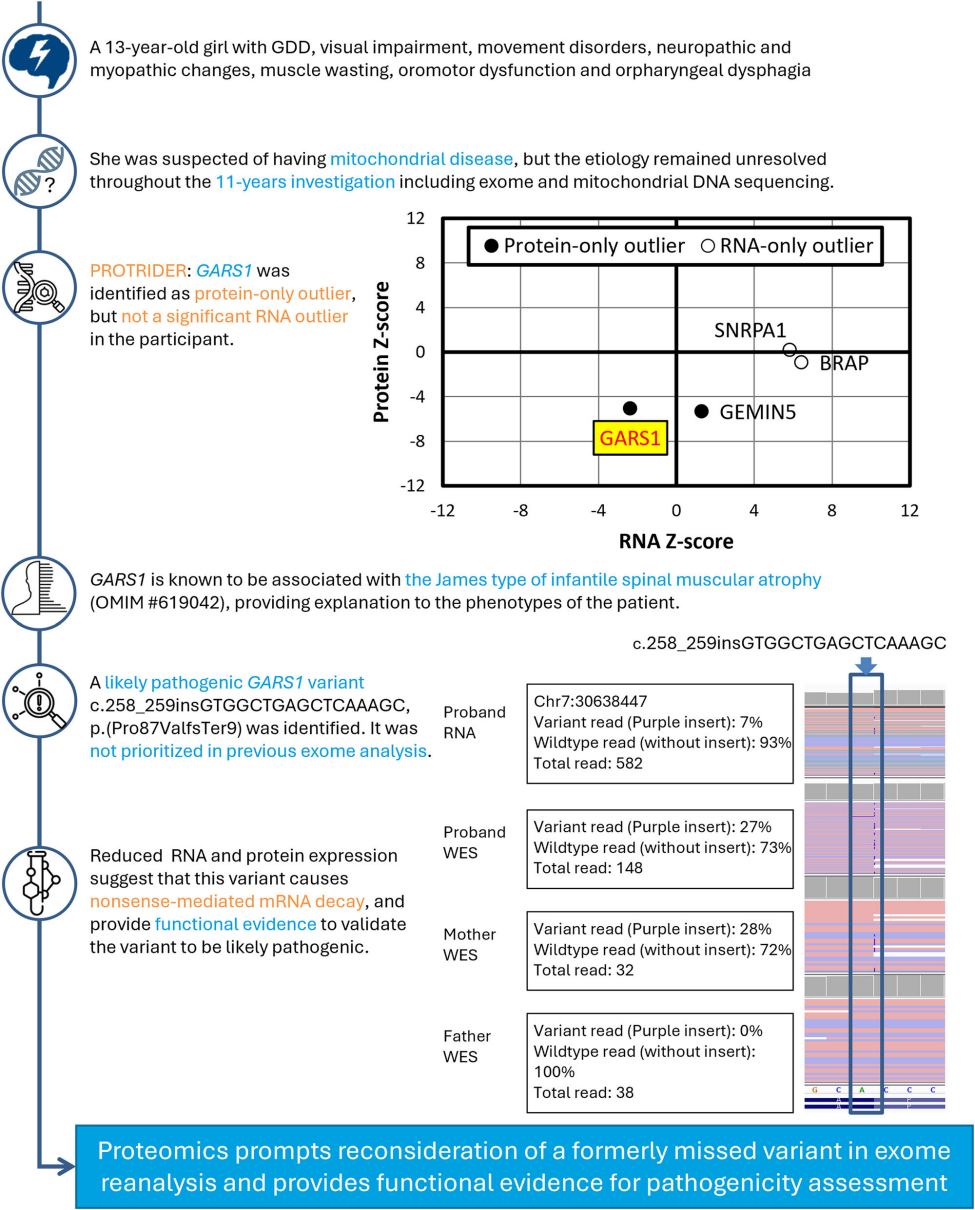
*GARS1* protein-only outlier in participant SF231. Multi-omics analysis suggests the reprioritization of 4 genes, including the protein-only outlier *GARS1*, in the exome reanalysis of the case. This gene was not detected as a significant RNA expression outlier (FDR > 0.1), yet with a fold change of 0.81 and a nominal *p*-value of 0.052. A likely pathogenic c.258_259insGTGGCTGAGCTCAAAGC; p.(Pro87ValfsTer9) insertion variant leading to a frameshift was then identified, which explains the individual’s phenotype. This variant was missed in the initial exome analysis.

The discovery of variants not previously prioritized by WES was guided by the detection of expression outliers in cases SF185, SF269, and SF231. In case SF185, a significant unbalanced expression (82%, FDR = 0.04) of a *MSTO1* missense variant was detected with the MAE pipeline, leading to the discovery of a splice region variant that was not prioritized by WES (Fig. 3). RNA-seq data further suggested that the splice region variant caused exon elongation and nonsense-mediated decay, which could account for the monoallelic expression of the missense variant. As for case SF269, a significant reduction in protein intensities was detected in SHMT2 (protein-only outlier: fold change = 0.37; Z-score = −5.92; FDR = 8.5 × 10^−5^), guiding the reprioritization of two compound heterozygous *SHMT2* missense variants suspected to affect protein stability, which only had limited evidence of pathogenicity in prior ES analysis (Fig. 4). In case SF231, the *GARS1* gene was detected as a protein-only outlier (fold change = 0.70; Z-score = −5.06; FDR = 0.011) in our preliminary trial with 40 samples, and a less significant result with nominal *p*-value of 0.00113 and FDR > 0.1 in final analysis. Despite a final FDR above our significance threshold, the initial finding indicating a notable reduction of fold change has prompted the reprioritization of a formerly missed *GARS1* insertion variant in WES curation (Fig. 5). Details of the curation process were illustrated in Supplementary Information 1.

**Fig. 6:**
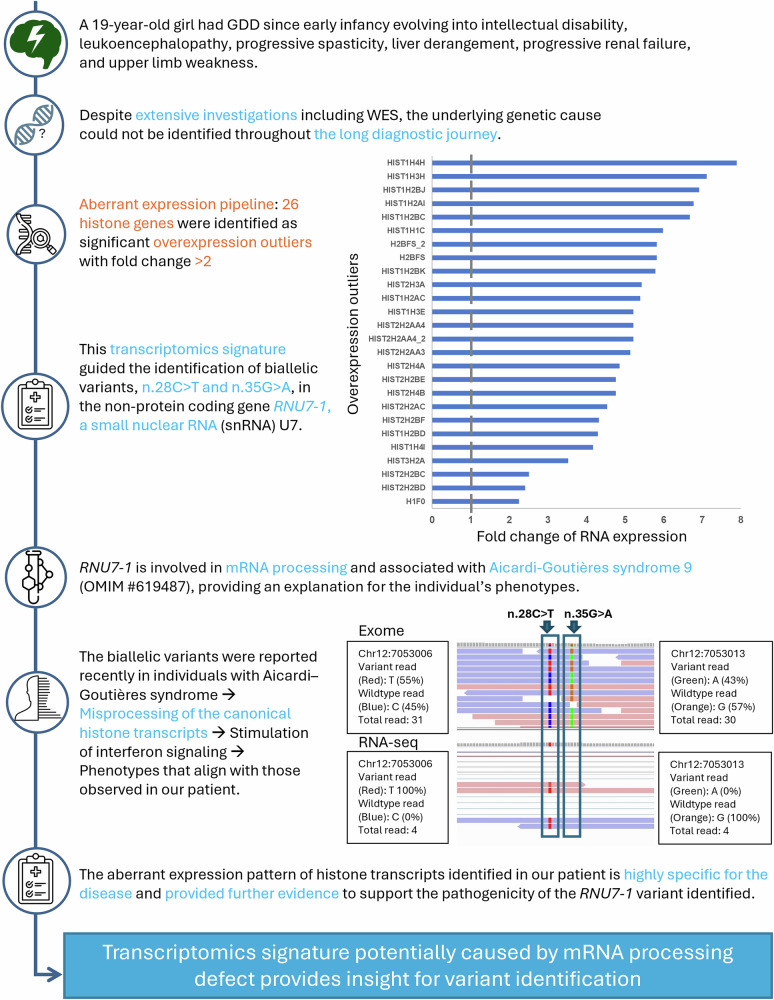
Identification of biallelic *RNU7-1* variants in participant SF188. Overexpression of 26 histone genes guided the identification of biallelic *RNU7-1* variants reported in individuals with Aicardi–Goutières syndrome, which resulted in misprocessing of the canonical histone transcripts, leading to phenotypes that align with those observed in our patient.

**Fig. 7:**
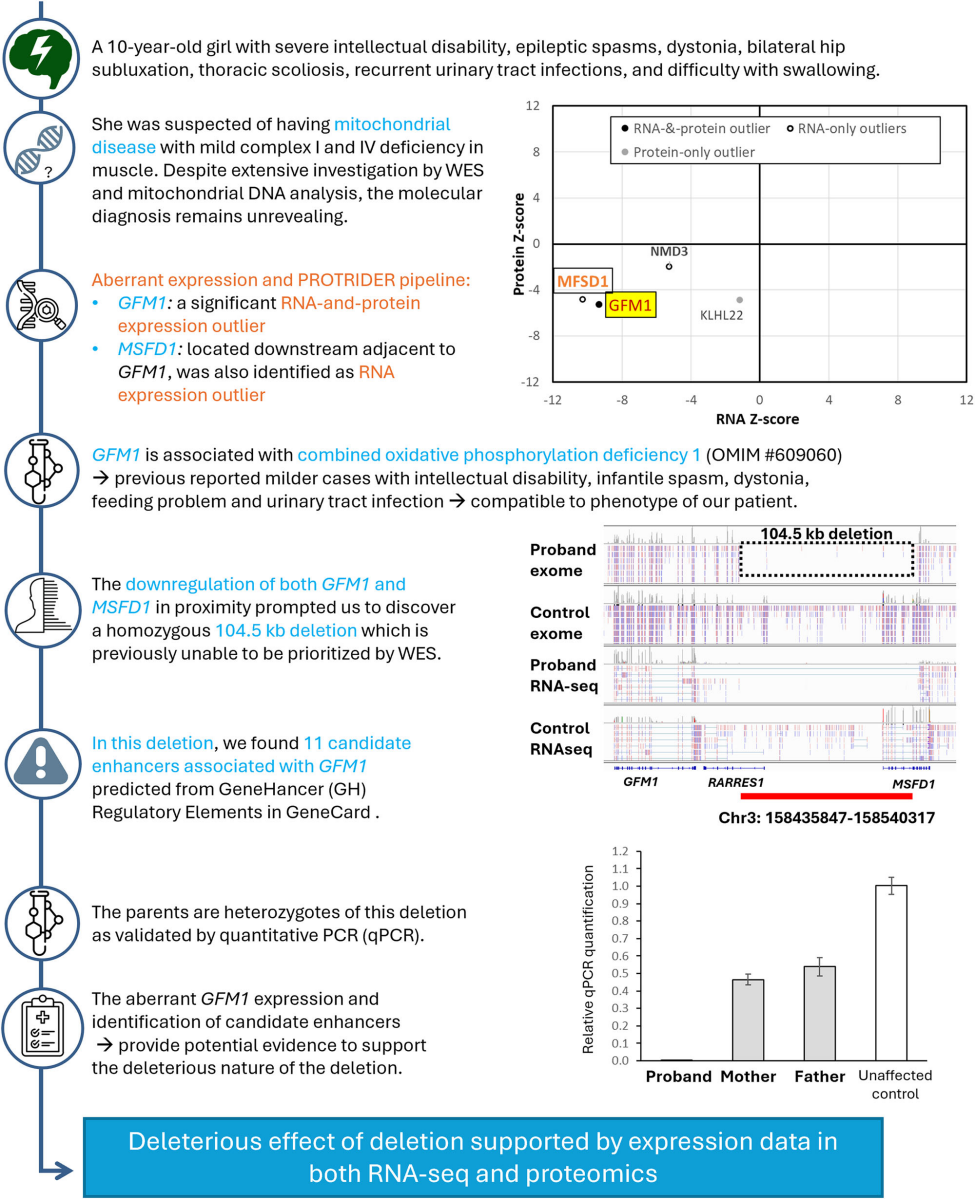
Identification of 104.5 kb deletion in participant SF197. Detection of expression outliers of *MSFD1* and *GFM1* guided the identification of a 104.5 kb deletion covering 11 candidate enhancers associated with *GFM1*, explaining the individual’s phenotypes.

Our outlier approach also uncovered phenotypes resulting from the causal variants, which led to the reprioritization of variants in cases SF188 and SF197. In case SF188, aberrant overexpression of 26 histone genes (2.86 × 10^−7^ ≤ FDR ≤ 0.054) guided re-examination of exome data to uncover compound heterozygous variants in *RNU7-1*, reported to impact canonical histone transcript processing in Aicardi–Goutières syndrome patients^35^, which was compatible with our participant’s presenting phenotype (Fig. 6). In case SF197, two genes in close proximity to each other were detected to have outlier RNA expression and outlier protein levels, *GFM1* (OUTRIDER: fold change: 0.35, Z-score = −9.76, FDR = 4.46 × 10^−15^; PROTRIDER: Z-score = −4.97, FDR = 0.017) and *MSFD1* (fold change 0.01, FDR = 1.39 × 10^−21^, Z-score = −11.38). WES re-examination revealed a homologous 100 kb deletion that was previously not prioritized, which led to the loss of 11 putative *GFM1* enhancers^36^ (Fig. 7; Supplementary Table 2).

Finally, 6 additional causal variants (in *FBXO28, GNB1, KCNT1, GABRG2, ATP1A3*, and *SPAST*) were identified and classified as likely pathogenic through exome reanalysis, with updated database and literature (Table 1). While 5 of those 6 genes are expressed in our fibroblast cohort (apart from *ATP1A3*), none of them were observed as RNA or protein outliers in these 6 cases. Also, 5 of those cases had missense variants that may alter protein functions but not affect RNA or protein levels significantly, and hence not assessed by our multi-omics approach for detecting expression outliers; whereas one case (SF074) involved a frameshift variant in the last exon, thus evading NMD. More detailed descriptions of these cases are included in Supplementary Information 1.

## Discussion

In this study, we utilized a comprehensive multi-omics approach, combining transcriptomics and proteomics with exome reanalysis, to enhance diagnostic capabilities in previously unresolved neurological diseases. We applied an outlier-detection approach to search for significantly aberrant RNA phenotypes and protein expression levels, using established computational workflows DROP^25^ and PROTRIDER^31^^,32^. The identified outliers were then used to prioritize candidate genes. We resolved 11 of the 34 cases analyzed (32%), with RNA-seq and proteomics playing a role in guiding the diagnosis in 5 cases (15%).

The strengths of a transcriptomic approach lie not only in its ability to locate the candidate gene via expression outliers, but also in the detection of RNA signatures. This was demonstrated by the overexpression of 26 histone genes in case SF188, which led to the discovery of causative *RNU7-1* variants linked to the misprocessing of these histone transcripts^35,37,38^. We were also able to detect changes in the noncoding regions, such as a deletion in overlapping candidate enhancers (case SF197). Other studies also demonstrate the ability of transcriptomics to identify the pathogenic RNA phenotypes that are the downstream effects of causal variants, when the casual variant was not detected in first-line DNA efforts^20,29,31^. This demonstrates the benefit of utilizing transcriptomics to detect abnormal events not easily interpreted or prioritized by DNA alone, with the additional possibility of revealing the underlying disease mechanism.

With the cost of multi-omics technologies decreasing rapidly and their speed and accessibility increasing, the integration of these omics into genetic diagnostics has become more feasible^39^. To date, studies on untargeted multi-omics for genetic diagnosis of rare diseases reported an increase in diagnostic rate from 7.5% to 36%^15–21,29,31^, but only two such published studies utilized proteomics^15,29^, and neither study utilized proteomics as an independent approach for guiding variant identification: Kremer et al.^15^ applied proteomics to investigate the impact of aberrant RNA levels found in 3 patients. Lunke et al.^29^ adopted proteomics for functional validation of an aberrant RNA expression in one patient. These ‘validation-only’ applications have not fully utilized the benefits of an untargeted proteomics protein-outlier approach. The tool PROTRIDER was released in 2021, in which the potential of a protein-outlier-detection approach for guided diagnoses was demonstrated, as the authors resolved 4 clinical cases using protein-only outliers^31^. In our study, protein-only outliers guided diagnosis in the reclassification of a missense variant based on decreased protein level in case SF269 and helped to detect a variant in case SF231, which was not previously prioritized in WES analysis. While our results are preliminary based on a small cohort, it is expected that proteomics will further help characterize variants that could not be prioritized by RNA expression levels.

Despite the potential of a protein-outlier approach to make diagnoses where DNA sequencing and RNA-seq have been unsuccessful, significant challenges remain. ACMG guidelines have not established the use of proteomics in variant classification^12^, and hence, prioritization based on this approach requires further interrogation. Updated recommendations on how to capture proteomics evidence regarding aberrant protein expression will be valuable in reclassifying these missense VUS, similar to the recent ACMG recommendations for RNA-assay evidence^40^. For example, in case SF269, we posit that the significant decrease in *SHMT2* protein level with a Z-score of −5.92 in the proteomics assay is an impact of the missense variant on protein stability. We then applied a threshold of Z-score ≤ -2, determined by a recent work using odds of pathogenicity (OddsPath) calculation as an initial framework for proteomics evidence^41^, to assign this SHMT2 finding as evidence for a strong level of pathogenicity (PS3). Apart from the suggested PS3 criteria, PP4, which considers the specificity of patient phenotype to the disease, might also be applied in proteomics-based reclassification, as patient-derived samples were used for assessment. However, that initial framework and our proposed suggestion would require more studies with larger cohorts, a broader range of diseases, and a variety of tissue types for the establishment of effective thresholds to become a solid guideline. Much work would be required for the future incorporation of proteomics-based evidence into ACMG guidelines. As for variants that only affect protein function but not protein stability, further functional characterization of mutant proteins would be necessary.

Another challenge in utilizing multi-omics for diagnosis is the coverage of disease-causing genes in tested tissues. Our findings showed that fibroblast transcriptomics and proteomics covered 71.1% and 53.2% of known neurological disease genes to complement the genetic analysis of undiagnosed rare diseases, while the detection of the remaining proportions could be hindered by our filtering criteria for well-expressed genes and the detection limits of the RNA-seq and proteomics protocols. Developmental stages and tissue-specific gene expression could also restrict the gene coverage, yet the genes not expressed in fibroblasts can be potentially studied through methods like transactivation of silent genes^42^, and transdifferentiation of patient fibroblasts into neuron-like cells^43,44^. These technologies are expected to further enhance the capability of multi-omics in the detection of molecular evidence for rare disease diagnosis.

Besides diagnosis, multi-omics can further aid the search for treatment options for rare disease patients. For example, transcriptomics can allow the exploration of antisense oligonucleotide (ASO) treatments to target RNA-specific phenotypes, such as modulating splice variants^45–47^, with an example of a clinical trial underway for developmental and epileptic encephalopathies (DEE)^48^. Aberrant RNA and protein expressions identified by multi-omics analyses could be extended to uncovering the disease-associated biological pathways, potentially for the drug development of pathway-targeting drugs. For instance, our multi-omics analyses guided the diagnosis of case SF188 with Aicardi–Goutières syndrome, who was affected by type I interferon-mediated autoinflammation. The syndrome has been putatively grouped into a family of disorders known as ‘interferonopathies’, which share a common pathological pathway of type I interferon signaling^49^. The shared disease mechanism enables the possibility of repurposing the drugs used in other interferonopathies for the treatment of AGS, such as JAK inhibitors^50,51^ (related clinical trial^52^: https://clinicaltrials.gov/study/NCT02974595), or reverse transcriptase inhibitors (related clinical trial^53^: https://clinicaltrials.gov/study/NCT02363452). Rasopathies^54–56^ and mTORopathies^57–59^ are other examples of using a repurposed drug for a group of rare disorders that share a common pathway, where patients with causative defects in different genes could still be treated with the same pathway inhibitor drug. Rare, orphan diseases often lack the funding or financial interest for the costly drug development process, but drug repurposing is a lower-cost method to find treatment options. Knowledge of the disease pathway is an essential precursor to identifying disease-specific treatments, and multi-omics could possibly aid in the precision medicine for rare diseases.

The incremental diagnostic yield, the added advantages in understanding the disease mechanism, and the potential of precision medicine laid a solid ground for the clinical implementation of multi-omics diagnosis. Many nationwide initiatives are exploring the multi-omics approach for rare diseases, where multi-omics-guided diagnoses were able to inform treatment decisions for individual patients.^29,60–63^ Our previous study estimated an average socio-economic cost of more than HKD 450,000 (approx. USD 58,000) per year per patient with rare neurologic disease, where earlier diagnosis rendered a reduction of costs and incidence of financial hardship^9^. The prompt diagnosis for rare diseases can be aided by our multi-omics pipeline, as demonstrated in this NDD cohort of 34 patients with a variety of clinical phenotypes and genetic causes. With our pipeline enabling the analysis of a diverse cohort at once, an establishment of a framework is required to further support its integration into the clinical workflow. For patients with candidate VUS identified in WES or WGS, we suggest recruiting them for multi-omics analysis if the candidate gene is expressed in fibroblasts with reference to public databases such as GTEx^33^. For other patients who remain undiagnosed after WES or WGS, we propose a case-by-case assessment by referral clinicians, clinical geneticists, and/or a multidisciplinary team. Based on clinical evaluation, patients would either be referred for multi-omics analysis or exome reanalysis after an interval in which the incorporation of reanalysis pipeline into the clinical protocol has been highlighted on multiple occasions, including in ACMG statements^11,64,65^. If exome reanalysis is negative, multi-omics analysis can be reconsidered. With the aid of appropriate workflows, more patients with rare diseases are expected to benefit from improved clinical management and precision medicine when multi-omics-guided diagnoses translate into prospective clinical practices.

In conclusion, our study highlights the potential of proteomics and transcriptomics to guide genetic diagnosis of neurological disorders. This approach holds promise not only for diagnosis, but also for understanding of disease mechanism, which has the potential of facilitating precision treatment and drug repurposing efforts.

## Methods

### Participants

Participants were recruited from the Queen Mary Hospital and the Hong Kong Children’s Hospital, which are hospitals affiliated with the University of Hong Kong (HKU). Individuals with unresolved NDDs in whom the underlying etiology remained unknown after standard clinical evaluation and WES analysis were recruited.

The study was approved by the institutional review board of the University of Hong Kong/Hospital Authority Hong Kong West Cluster and Hong Kong Children Hospital (UW11-190, UW12-211, and HKCH-REC-2020-032a), and complied with all relevant ethical regulations, including the Declaration of Helsinki. Written informed consents were obtained from all participants or their guardians.

### Patient fibroblast culture and RNA sequencing

Primary fibroblast cell lines were derived from participants’ skin biopsies and cultured in high-glucose Dulbecco’s Modified Eagle Medium (DMEM) supplemented with 10% fetal bovine serum and 1% penicillin/streptomycin (Thermo Fisher Scientific, Waltham, MA). RNA extraction, RNA-seq, and the subsequent data processing and analysis were performed as described in our previous transcriptomics study^66^. As sequencing depth correlates with the sensitivity of detecting aberrant RNA outliers^22,25,23^, RNA was sequenced at a 100 million read depth.

### LC‒MS-based quantitative proteomics

Protein samples were processed for LC‒MS/MS analysis performed at the Centre for PanorOmic Sciences at the University of Hong Kong (HKU). Proteins were extracted from skin fibroblasts, and then reduced, alkylated, and digested by Lys-C (FUJIFILM Wako Chemicals, US) for 3 h at 25 °C and by trypsin (Promega, Madison, US) overnight at 25 °C. Digests were acidified, desalted, and Tandem Mass Tag (TMT)-labeled by 11-plex TMT reagents (Thermo Fisher Scientific) following the manufacturer’s instructions. TMT-labeled samples were fractionated sequentially using a Peptide BEH C18 column, and 8 fractions were collected for each sample. The LC system was directly coupled in-line with an Orbitrap Fusion Lumos mass spectrometer (Thermo Fisher Scientific), which was operated in a data-dependent acquisition mode for the MS2 analysis. The raw MS data were processed using Proteome Discover 3.0 software and searched against the Human UniProt FASTA database. Confident proteins were identified using a target-decoy approach using a reversed database with FDR < 1%. A supplementary statistical workflow, MSFragger, was applied as a peptide identification and quantification tool^67^.

### Defining and filtering expressed genes covered by RNA-seq and proteomics

For RNA-seq, the expressed genes were defined as genes for which at least 5% of the samples had an FPKM > 1, as suggested by OUTRIDER^22^. For the protein expression, we selected proteins with a detectable intensity in at least 70% of the samples. The filtered expressed genes are used for gene-panel coverage and multi-omics analysis.

### Detection of aberrant RNA outliers using DROP

DROP workflow has been applied for the detection of AE, AS, and MAE outliers in an outlier approach as described in previous studies^19^^,20^. AE was detected by OUTRIDER^22^, and significant AE events were defined as those with a false discovery rate (FDR) ≤ 0.1 after correcting for multiple testing. While previous studies used a threshold of FDR ≤ 0.05^19,20^, we decided to loosen the threshold to prevent missing any events caused by potential pathogenic variants. AS was detected using the improved FRASER 2.0 algorithm^24^, which has a new intron-based metric, Intron Jaccard Index (Δ*J*), that represents the proportion of reads that support the splicing of a specific intron among all reads related to both splice sites of the intron. Significant AS events were defined with cutoffs of FDR ≤ 0.1 and absolute effect size |*J* | ≥ 0.1. MAE was identified by the MAE module of DROP, with the counting of the reads aligned to each allele at genomic positions of heterozygous variants from WES data as described in previous studies^20,25^. 1 study participant did not undergo exome sequencing, and therefore MAE was not run for this case (SF285) (Supplementary Data 1). A cutoff of allele ratio ≥0.8 and FDR < 0.05 was used to classify a variant as mono-allelically expressed.

### Detection of protein outliers

Aberrant protein expression was detected as previously described^31^. Significant protein expression outliers were detected by PROTRIDER^31,32^, defined as those with FDR ≤ 0.1.

### Exome reanalysis, variant identification, interpretation, and validation

Of the variants and outliers detected, those that are reported as disease-associated genes in Online Mendelian Inheritance in Man (OMIM) were prioritized for manual inspection. The genes were further prioritized if they had phenotypes and modes of inheritance that matched with clinical presentations. Causative variants of outlier genes were searched from WES and RNA-seq data. The WES data were re-annotated using the most up-to-date databases in Geneyx Analysis platform v5.15 (Geneyx Genomex Ltd., Israel)^68^ at the time of analysis, and manual curation of variants was performed concerning the genotype-phenotype correlation in each case, allele frequency (<1% in gnomAD v.2.1.1), inheritance pattern, and in silico predictions. The pathogenicity of variants was interpreted based on the American College of Medical Genetics and Genomics and the Association for Molecular Pathology (ACMG/AMP) guideline^69^ and the recently published framework to capture evidence based on RNA-assay evidence^40^.

### Gene variant confirmation with Sanger sequencing and quantitative PCR

Genomic DNA was extracted from fibroblasts (for proband’s DNA) using QIAamp® DNA Mini Kit (Qiagen GmbH, Germany) and peripheral blood (for parents’ DNA) using Flexigene DNA Kit (Qiagen GmbH, Germany). Quality of the genomic DNA was evaluated by agarose gel analysis, and quantity was measured by Qubit® dsDNA assay (Thermo Fisher Scientific, Waltham, MA). Primers were ordered from Integrated DNA Technologies (Coraville, IA). A full list of primers used is included in Supplementary Table 1.

For confirmation of variants identified in RNA-seq, Sanger sequencing was performed for variant confirmation and segregation analysis for the singleton cases. Forward and reverse primers flanking the variants according to the genomic reference sequence (GRCh37/hg19) were designed (Supplementary Table 1). Sanger sequencing reactions were run on Applied Biosystems^TM^ 3730xl DNA Analyzer (Thermo Fisher Scientific, Waltham, MA) at the Centre for PanorOmic Science at HKU.

Sanger sequencing was used to determine the breakpoints of the deletion in sample SF197. Primers SF197-BPf and SF197-BPr (Supplementary Table 1) were designed near each putative breakpoint region as upstream forward primer and downstream reverse primer, respectively, using the genomic reference sequence GRCh37/hg19. PCR amplification was performed in the proband, parents, and control samples. PCR products were then sequenced by Sanger sequencing, and sequencing results were aligned with the reference genome using BLAT of UCSC Genome Browser (https://genome.ucsc.edu/cgi-bin/hgBlat) for mapping breakpoints at the single-nucleotide level.

Quantitative PCR (qPCR) was performed on the proband, parents, and negative control for validation of the deletions in SF197. Primers SF197-RT-F and SF197-RT-R were designed as forward and reverse primers, respectively, according to the genomic reference sequence (GRCh37/hg19) of the deleted region. Real-time PCR reaction was performed using DNA extracted from the peripheral blood of the patient, mother, father, and normal control on Roche LightCycler® 480 system (Roche Diagnostics, Rotkreuz, Switzerland) using TB Green Premix Ex Taq^TM^ Tli RNaseH Plus (Takara Bio Inc., Shiga, Japan) according to the manufacturer instructions. The number of copies of each sample was determined by the ΔΔ*Ct* method and compared the *Ct* (cycle threshold) in the proband and parents with that in the normal control. Three independent experiments were performed. Melting curve analysis was performed to ensure the specificity of the PCR amplification.

## Supplementary information

Supplementary Information

Supplementary Information

## Data Availability

The datasets generated and analyzed during the current study are not publicly available due to ethical concerns regarding minor participants and patient anonymity. Request to access the datasets can be directed to the corresponding author, and data will be de-identified before they are transmitted to qualified researchers.
