# Supplementary material for: An outlier approach: advancing diagnosis of neurological diseases through integrating proteomics into multi-omics guided exome reanalysis
Source: NPJ Genom Med. 2025 May 3;10:36. doi: 10.1038/s41525-025-00493-5 (PMC12049463; doi:10.1038/s41525-025-00493-5)
Supplement: Supplementary file 1 — Supplementary Information [file 41525_2025_493_MOESM1_ESM.pdf]

Supplementary Information for the manuscript "An outlier approach: Advancing diagnosis of neurological diseases through integrating proteomics into multi-omics guided exome reanalysis"

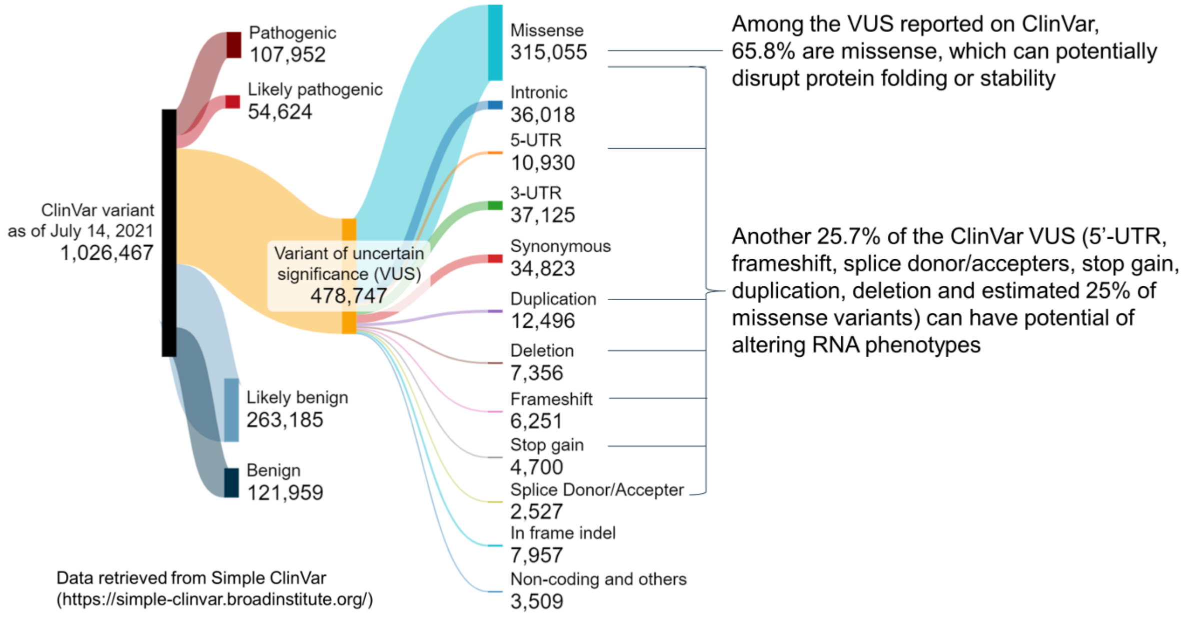

Supplementary Figure 1. Sankey diagram depicting the distribution of variants of uncertain significance (VUS) reported in ClinVar database as of July 14, 2021. The proportion of missense variants having potential RNA phenotypes was estimated from a recent study (Smirnov D et al. Hum Mutat. 2022 Aug;43(8):1056-1070.). Data retrieved from Simple ClinVar (Pérez-Palma E et al. Nucleic Acids Res. 2019 Jul 2;47(W1):W99-W105) on 10th March, 2025 at https://simple-clinvar.broadinstitute.org/

Supplementary Information 1: Details of the 11 participants that were diagnosed by further exome reanalysis

SF074

The participant presented with GDD, microcephaly, feeding problems, and failure to thrive. Besides, he displayed movement disorders characterized by dystonia and chorea. Encephalopathy associated with respiratory syncytial virus infection was suspected at 7 months old, followed by the development of epileptic spasms with varying seizure control. The participant also exhibited brainstem dysfunction in the form of central hypoventilation. Additional clinical findings encompassed peripheral neuropathy, lower limb hypotonia, areflexia, and bilateral optic atrophy. Progressive loss of white matter on MRI was also noted.

By exome reanalysis, a likely pathogenic *FBXO28* (NM_015176.4): c.1073_1074del, p.(Leu358GlnfsTer4) variant was identified (ACMG/AMP evidence: PM1, PM4, PM2_supporting and PM6_supporting). *FBXO28* variant is associated with developmental and epileptic encephalopathy (DEE) 100 (MIM number: 619777), a severe neurological disorder with GDD, early onset of variable types of refractory seizures, profoundly impaired intellectual development, and movement disorders. It is a frameshift variant in the last exon predicted to cause truncation at the C-terminal region and the transcript is predicted to escape nonsense-mediated decay. Frameshift *FBXO28* variant was first discovered in 2018[1] and a recent study reported *FBXO28* variants in 10 unrelated patients with DEE100[2]. In that study, several truncating variants were identified in positions 325-360 of the protein, and a DNA level variant was identified in this region that led to a truncation at position 362. They showed that a more severe phenotype was associated with truncating variants compared to missense variants. This new finding suggested that this position is a mutational hotspot with the truncating variants associated with severe phenotypes and provided further evidence for

the variant classification.

SF108

This was a 21-year-old male characterized by GDD and short stature that has progressed to moderate intellectual disability. The participant initially displayed hypotonia during infancy, which later transitioned into hypertonia. Progressive muscle weakness, spasticity, and progressive dystonia were subsequently observed. Additionally, he was presented with bilateral sensorineural deafness and bilateral undescended testes. Metabolic workup revealed endocrine dysfunction with cortisol insufficiency. Further investigations unveiled a reduced mitochondrial DNA/nuclear DNA (mtDNA/nDNA) ratio, indicating the possibility of mtDNA depletion syndrome.

A likely pathogenic missense *GNB1* (NM_002074.5): c.239T>C, p.(Ile80Thr) variant was identified by exome reanalysis (ACMG/AMP evidence: PS4_moderate, PM1, PM5 and PM2_supporting). *GNB1* encodes a β subunit of heterotrimeric G proteins for the signaling of G-protein-coupled receptors and is associated with intellectual developmental disorder 42 (MIM number: 616973) characterized by GDD, impaired intellectual development and hypotonia which is consistent with the main clinical feature of the participant. Recurrent variants at the same amino acid position could be identified in more than 10 patients with spasticity, dystonia, undescended testes, and sensorineural hearing loss in studies in 2016 and 2018[3,4] and provided further evidence to support the pathogenicity of the variant. Deep brain stimulation was reported to have remarkable initial benefit to myoclonus-dystonia resulting from the *GNB1* variants[5]. Moreover, this finding suggested that mtDNA depletion is not the primary cause of the patient's phenotype but a secondary manifestation.

SF113

This case was a 14-year-old male with profound intellectual disability and a lack of significant developmental progress. He was presented with refractory focal seizures, feeding difficulties, and cortical visual impairment. Upon physical examination, he displayed generalized hypotonia, spastic quadriplegia, and significant scoliosis. Muscle mitochondrial DNA (mtDNA) analysis revealed a reduced mtDNA/nuclear DNA (nDNA) ratio, suggestive of mtDNA depletion.

A likely pathogenic variant, NM_020822.2: c.1546A>G, p.(Met516Val), was identified by exome reanalysis in the *KCNT1* gene (ACMG/AMP evidence: PM6_strong, PS4_moderate, PM2_supporting) encoding the subunit of Na^{+}-activated K^{+} channels, and associated with DEE14 (MIM number: 614959), manifested as malignant Migrating Partial Seizures in Infancy (MMPSI). DEE14 is characterized by early-onset intractable focal seizures with neurodevelopment arrest, hypotonia and quadriplegia[6] which is compatible with the phenotype. Previous study in 2016 showed that this M516V variant causes a gain-of-function effect on *KCNT1* channels and the use of *KCNT1* blockers quinidine can be a possible treatment for this *KCNT1*-related epileptic disorder[7]. After this functional characterization, the same missense variant has been reported in other patients with the same phenotypes[8,9] and he functional study and the subsequent reports provide further evidence to support the pathogenicity of the variant. Again, our present finding suggested that mtDNA depletion is a secondary manifestation of the disease.

SF171

This was a 19-year-old male with GDD and severe intellectual disability. At 15 months old,

he began experiencing multiple types of seizures, including myoclonic, tonic, febrile, afebrile, and multifocal seizure onset. Ophthalmoplegia, visual impairment, and ptosis were also evident. Additional observations included myopathy with facial involvement and generalized dystonia, resulting in the patient being wheelchair-bound. An electroencephalogram (EEG) revealed frequent runs of interictal spike discharges associated with epileptic encephalopathy, providing further insights into the participant's condition.

A likely pathogenic missense *GABRG2* (NM_000816.3): c.316G>A,p.(Ala106Thr) variant was identified (ACMG/AMP evidence: PS4_moderate, PM6_moderate, PM2_supporting, PP2) to be associated with DEE74 (MIM number: 618396) characterized by early onset of intractable seizures with variable seizure types, severe GDD with hypotonia, motor impairment and abnormal eye movements which is compatible with our participant's clinical features. A previous study demonstrated that the A106T variant could affect the $GABA_A$ receptor function and reduce $GABA_A$ current, and different EEG abnormalities could be identified in the patients[10]. After the report of this functional study, the same missense variant has been reported in several persons with the same phenotypes[10–14]. The functional characterization and the subsequent reports of the same variant in different individuals provided additional evidence to support the pathogenicity based on the ACMG/AMP framework.

SF180

This was a 39-year-old male who exhibited GDD and central hypotonia at 13 months of age. He subsequently developed muscle weakness in both upper and lower limbs, accompanied by spasticity in the lower limbs. Over time, his condition deteriorated, resulting in progressive lower limb weakness and near-complete paralysis, along with associated urinary and bowel

control issues.

A likely pathogenic *ATP1A3* (NM_152296.5): c.2324C>T, p.(Pro775Leu) variant was identified (ACMG/AMP evidence: PM2_supporting, PS4_moderate, PS3_supporting, PM5 and PM6_supporting). *ATP1A3* encodes the sodium-potassium ATPase α3 catalytic subunit exclusively expressed in neurons. *ATP1A3* genetic defects were associated with a spectrum of *ATP1A3*-related neurological syndromes. A recent study in 2023 identified a novel recurrent *ATP1A3* variant in 9 persons which is the same variant found in this study. They were presented with progressive or nonprogressive spasticity, DD and intellectual disability but they were initially not suspected to have *ATP1A3*-related disorder as their phenotypes did not meet the diagnostic criteria of these neurological syndromes[15]. Functional study by ouabain complementation assay demonstrated that this P775L variant could cause loss-of-function of α3-Na+/K+-ATPase and introduce inward current leak[15]. This new finding provides additional evidence to support the pathogenicity of this variant that correlates with a milder phenotype resembling idiopathic spastic cerebral palsy and hereditary spastic paraplegia.

SF185

This was a 12-year-old boy who exhibits global developmental delay (GDD), progressive cerebellar ataxia that began at 31 months, progressive cerebellar atrophy and progressive proximal myopathy. He was suspected of having mitochondrial disease, but the etiology remained unresolved throughout the 10-year investigation. Through the MAE pipeline, *MSTO1* transcripts with a reported likely pathogenic missense mutation [NM_018116.4: c.971C>T, p.(Thr324Ile)] were detected to have an significant unbalanced expression (82%, FDR = 0.0398). This gene is associated with autosomal recessive mitochondrial myopathy and ataxia (MIM number: 617675), consistent with the individual's phenotypes. A splice

region variant c.967-3C>A was identified in another allele at 3 bp into the intron that cannot be prioritized by ES initially as it is not located in the direct splice site. Our RNA-seq data demonstrated that this unreported splice region variant causes exon elongation with putative starting point at c.966+65, which was not detected as AS outlier (FDR>0.1) but hinted by a nominal p-value of 0.0258 and an effect size ΔJ of -0.2 in FRASER results. The affected transcript was predicted to undergo NMD, as there was a premature termination codon at c.966+104 and a slightly reduced transcript expression (OUTRIDER: fold change = 0.71, Z score = -4.13, nominal p-value = 1.13 $x10^{-4}$, FDR = 1), leaving another allele to have monoallelic expression. This functional evidence classified this splice region variant as likely pathogenic.

SF188

This 19-year-old girl had GDD since early infancy evolving into intellectual disability, leukoencephalopathy, progressive spasticity, liver derangement, progressive renal failure, and upper limb weakness. Despite extensive investigations including ES, the underlying genetic cause could not be identified throughout the long diagnostic journey. In our AE pipeline, 26 histone genes were identified as significant overexpression outliers with fold change ranging from 2.25-7.89 (FDR= $2.86x10^{-7}$-0.054). This transcriptomics signature guided further investigation of ES data, leading to the identification of two likely pathogenic biallelic variants, n.28C>T and n.35G>A, in the non-protein coding gene *RNU7-1*. It is a small nuclear RNA (snRNA) U7 which is involved in mRNA processing, and associated with Aicardi-Goutières syndrome 9 (MIM number: 619487), providing an explanation for the individual's phenotypes. The biallelic variants, which were recently reported in several individuals with Aicardi–Goutières syndrome, resulted in misprocessing of the canonical

histone transcripts and stimulation of interferon signaling[16], ultimately leading to phenotypes that align with those observed in our patient. The aberrant expression pattern of histone transcripts identified in our patient is highly specific for the disease, and upgraded the PP4 rule to a strong level along with further molecular evidence to support the pathogenicity of the *RNU7-1* variant identified.

SF196

This was a 12-year-old boy with multiple conditions including GDD affecting both motor skills and speech, mild intellectual disability, autism spectrum disorder, muscle weakness, mild spasticity, and dystonia. Spasticity is more pronounced in the lower limbs, leading to difficulty in walking.

A likely pathogenic variant has been identified in the *SPAST* gene (NM_014946.4): c.1385A>G, p.(Lys462Arg) (ACMG/AMP evidence: PM2_supporting, PP3_moderate, PM1, PM5). Variants in this gene are associated with spastic paraplegia type 4 (MIM number: 182601), a condition characterized by muscle weakness, progressive muscle spasticity in the legs, and walking difficulty. This variant alters the structure or disrupts the production of spastin, which is encoded by *SPAST*. As a result, the ability of spastin to regulate microtubules is impaired. Microtubules are responsible for transporting organelles in nerve cells, and this disruption leads to the symptoms associated with spastic paraplegia type 4. The identified variant is located in the ATPases Associated with diverse cellular Activities (AAA) domain, which is crucial for microtubule severing and is enriched with *SPAST*-related pathogenic variants[17]. The variant was not prioritized by initial ES due to limited evidence but was now classified as likely pathogenic because variants with a different missense change at the same amino acid residue were further reported in ClinVar and latest recommendation

for ACMG/AMP classification, which involves the use of in silico tools, has been released[18].

SF197

A 10-year-old girl presented with various symptoms including severe intellectual disability, epileptic spasms, dystonia, bilateral hip subluxation, thoracic scoliosis, recurrent urinary tract infections, and difficulty with swallowing. She was suspected of having mitochondrial disease with mild complex I and IV deficiency in muscle. Despite extensive investigation by ES and mitochondrial DNA analysis, the molecular diagnosis remains unrevealing. In this study, the *GFM1* gene was identified as a significant RNA-and-protein outlier (OUTRIDER: fold change = 0.35, Z score = -9.76, FDR = 4.46×10^{-15}; PROTRIDER: Z score = -4.97, FDR = 0.017), indicating >50% reduction in expression, comparable to the effect of homozygous variants as reported[19,20]. The phenotype can be explained by the association of *GFM1* with combined oxidative phosphorylation deficiency 1 (MIM number: 609060). Previous studies reported some milder cases with intellectual disability, infantile spasm, dystonia, feeding problem and urinary tract infection which are compartible[21–23]. *MSFD1,* located downstream adjacent to *GFM1*, was also identified as AE outlier (fold change 0.01, FDR = 1.39×10^{-21}, Z-score = -11.38). The downregulation of two genes in proximity prompted us to investigate and ultimately discover a homozygous 104.5 kb deletion (chr3:158435847-158540317) covering *MSFD1*, which is previously unable to be prioritized by ES. The parents are heterozygotes of this deletion. In this region, 11 candidate enhancers associated with *GFM1,* which is 77.6-174.8 kb downstream from the *GFM1* transcription start site, were predicted from GeneHancer (GH) Regulatory Elements in GeneCard (Supplementary information 4)[24]. The aberrant *GFM1* expression and identification of candidate enhancers in the noncoding deleted region provide potential evidence to support the deleterious nature of the deletion.

SF231

In a participant with GDD, visual impairment, movement disorders, neuropathic and myopathic changes, muscle wasting, oromotor dysfunction and orpharyngeal dysphagia, GARS1 was identified as significant protein-only outliers in initial PROTRIDER run using 40 proteomics data (fold change=0.697; Z score = -5.06; FDR= 0.011) but it was detected with p-value = 0.001127 but FDR > 0.1 in final run with 60 samples. It was not detected as significant RNA expression outliers (fold change=0.81, p-value=0.052) all along. *GARS1* is associated with infantile James type spinal muscular atrophy inherited in an autosomal dominant manner (MIM number: 619042), providing explanation to the phenotype. A likely pathogenic *GARS1* variant [c.258_259insGTGGCTGAGCTCAAAGC, p.(Pro87ValfsTer9)] was identified. Previously, this variant was not prioritized in the ES. However, the abnormal protein expression of GARS1, as revealed by proteomics, prompted a reconsideration of its significance as a finding worth reporting in this context.

SF269

A 16-year-old boy presented with intrauterine growth restriction, dysgenesis of corpus callosum, microcephaly, mild intellectual disability, dystonia, spasticity, dysmorphic features, intermittent drooling, and autism spectrum disorder. His younger sister has developed similar phenotypes. Despite previous unceasing investigations involving ES and subsequent reanalysis, the siblings' phenotypes remained unexplained. In this study, SHMT2 was identified as protein-only outliers (fold change = 0.369; protein outlier Z score = -5.92; FDR= 8.50×10^{-5}), but not as RNA outliers in the participant. Subsequently, two compound heterozygous *SHMT2* missense variants c.1042C>T, p.(R348W) and c.1301G>A, p.(R434Q) were identified in the siblings. *SHMT2* is known to be associated with neurodevelopmental

disorders with cardiomyopathy, spasticity, and brain abnormalities (MIM number: 619121). The patients previously reported with biallelic SHMT2 variants exhibited all clinical features consistent with the sibling's phenotypes[25]. Our data indicated that these missense *SHMT2* variants may destabilize proteins, providing functional evidence to support the pathogenicity of the variants, which were previously difficult to prioritize using ES due to limited evidence.

Supplementary Table 1: List of primers and their sequences

Primer name	Sequence
SF197-BP-F	AGTCTTGCTCTGTCGCCC
SF197-BP-R	TGTTAAATACGCAAACCCACTG
SF197-RT-F	ACTGGCAATCAAGAAAGTGGAC
SF197-RT-R	TCAGTTTCTTCACAGACCTCCA
SF188-F	TCGCGAACTCTAGAAATGAATG
SF188-R	CTAGGACGATTCTCACCCTTTG
SF074-F	GAACAAAATGCACGGTTGGC
SF074-R	GCCTAGAAGCTTAAGGGACAC
SF113-F	CTGGGAGAGAAGCACAGGAG
SF113-R	CTTCCCCTGGCATGGATCC
SF171-F	AAAATCTTGCACCTCTCTATGTG
SF171-R	TCTGGAAGACTATCTTTCACTTCTAAA
SF180-F	AAAAGGACGTTGGATGAGGGC
SF180-R	AGTGAGATGGCAGGGACCTAG
Legend: F – Forward. R – Reverse.	

Supplementary Table 2: Candidate enhancers for GFM1 predicted be affected in patient SF197 based on GeneHancer (GH) database[24] in the framework of GeneCards

GeneHancer identifier	GH type	Genomic Location (GRCh37/hg19)	Enhancer confidence score (GH score)*	Gene-Enhancer association score#	Distance downstream (kb)
GH03J158715	Enhancer	chr3:158433310-158446330	1.6	22.6	77.5
GH03J158742	Enhancer	chr3:158459790-158469746	1.3	18.7	102.5
GH03J158731	Promoter/ Enhancer	chr3:158448989-158451990	1.8	14.6	88.2
GH03J158767	Enhancer	chr3:158484990-158497389	1.7	7.0	128.9
GH03J158791	Promoter/ Enhancer	chr3:158509221-158509370	0.9	11.2	147.0
GH03J158800	Promoter/ Enhancer	chr3:158518589-158522590	1.9	5.2	158.3
GH03J158792	Enhancer	chr3:158509823-158511369	0.6	11.2	148.3
GH03J158781	Enhancer	chr3:158498990-158500789	0.8	7.0	137.6
GH03J158788	Enhancer	chr3:158506027-158507110	0.5	7.0	144.3
GH03J158817	Enhancer	chr3:158535391-158538788	0.4	7.2	174.8
GH03J158754	Enhancer	chr3:158469790-158473988	0.3	7.2	109.6

*Enhancer confidence score represents the degree of confidence of each enhancer[24].

Gene-Enhancer association score was calculated for each gene–enhancer link, to estimate the strength of such connection[24].

References:

1. Balak, C. *et al.* A novel FBXO28 frameshift mutation in a child with developmental delay, dysmorphic features, and intractable epilepsy: A second gene that may contribute to the 1q41-q42 deletion phenotype. *Am J Med Genet A.* **176**, 1549–1558 (2018).
2. Schneider, A. L. *et al.* FBXO28 causes developmental and epileptic encephalopathy with profound intellectual disability. *Epilepsia* **62**, e13–e21 (2021).
3. Hemati, P. *et al.* Refining the phenotype associated with GNB1 mutations: Clinical data on 18 newly identified patients and review of the literature. *Am J Med Genet A.* **176**, 2259–2275 (2018).
4. Petrovski, S. *et al.* Germline De Novo Mutations in GNB1 Cause Severe Neurodevelopmental Disability, Hypotonia, and Seizures. *Am J Hum Genet.* **98**, 1001–1010 (2016).
5. Jones, H. F. *et al.* Myoclonus-dystonia caused by GNB1 mutation responsive to deep brain stimulation. *Mov Disord.* **34**, 1079–1080 (2019).
6. Coppola, G., Plouin, P., Chiron, C., Robain, O. & Dulac, O. Migrating partial seizures in infancy: a malignant disorder with developmental arrest. *Epilepsia* **36**, 1017–1024 (1995).
7. Rizzo, F. *et al.* Characterization of two de novoKCNT1 mutations in children with malignant migrating partial seizures in infancy. *Mol Cell Neurosci.* **72**, 54–63 (2016).
8. Barcia, G. *et al.* Epilepsy with migrating focal seizures: KCNT1 mutation hotspots and phenotype variability. *Neurol Genet.* **5**, e363 (2019).
9. Numis, A. L. *et al.* Lack of response to quinidine in KCNT1-related neonatal epilepsy. *Epilepsia* **59**, 1889–1898 (2018).
10. Shen, D. *et al.* De novo GABRG2 mutations associated with epileptic encephalopathies. *Brain* **140**, 49–67 (2017).

11. Kim, M.-J., Yum, M.-S., Seo, G. H., Ko, T.-S. & Lee, B. H. Phenotypic and Genetic Complexity in Pediatric Movement Disorders. *Front Genet.* **13**, 829558 (2022).
12. Seo, G. H. *et al.* Diagnostic performance of automated, streamlined, daily updated exome analysis in patients with neurodevelopmental delay. *Mol Med.* **28**, 38 (2022).
13. Yang, Y. *et al.* Phenotypic Spectrum and Prognosis of Epilepsy Patients With GABRG2 Variants. *Front Mol Neurosci.* **15**, 809163 (2022).
14. Zou, F. *et al.* Expanding the phenotypic spectrum of GABRG2 variants: a recurrent GABRG2 missense variant associated with a severe phenotype. *J Neurogenet.* **31**, 30–36 (2017).
15. Calame, D. G. *et al.* Cation leak through the ATP1A3 pump causes spasticity and intellectual disability. *Brain* **146**, 3162–3171 (2023).
16. Uggenti, C. *et al.* cGAS-mediated induction of type I interferon due to inborn errors of histone pre-mRNA processing. *Nat Genet.* **52**, 1364–1372 (2020).
17. Shoukier, M. *et al.* Expansion of mutation spectrum, determination of mutation cluster regions and predictive structural classification of SPAST mutations in hereditary spastic paraplegia. *Eur J Hum Genet.* **17**, 187–194 (2009).
18. Pejaver, V. *et al.* Calibration of computational tools for missense variant pathogenicity classification and ClinGen recommendations for PP3/BP4 criteria. *Am J Hum Genet.* **109**, 2163–2177 (2022).
19. Murdock, D. R. *et al.* Transcriptome-directed analysis for Mendelian disease diagnosis overcomes limitations of conventional genomic testing. *J Clin Invest.* **131**, (2021).
20. Yépez, V. A. *et al.* Clinical implementation of RNA sequencing for Mendelian disease diagnostics. *Genome Med.* **14**, 38 (2022).
21. Brito, S. *et al.* Long-term survival in a child with severe encephalopathy, multiple respiratory chain deficiency and GFM1 mutations. *Front Genet.* **6**, 102 (2015).

22. Simon, M. T. *et al.* Activation of a cryptic splice site in the mitochondrial elongation factor GFM1 causes combined OXPHOS deficiency. *Mitochondrion* **34**, 84–90 (2017).
23. Glasgow, R. I. C. *et al.* Novel GFM2 variants associated with early-onset neurological presentations of mitochondrial disease and impaired expression of OXPHOS subunits. *Neurogenetics* **18**, 227–235 (2017).
24. Fishilevich, S. *et al.* GeneHancer: genome-wide integration of enhancers and target genes in GeneCards. *Database (Oxford)* **2017**, (2017).
25. García-Cazorla, À. *et al.* Impairment of the mitochondrial one-carbon metabolism enzyme SHMT2 causes a novel brain and heart developmental syndrome. *Acta Neuropathol.* **140**, 971–975 (2020).
